# *N*,1-Bis(4-chloro-2-methyl­benz­yl)-3-methyl-2-oxo-1,2,3,4-tetra­hydro­quinoline-3-carboxamide

**DOI:** 10.1107/S1600536809046765

**Published:** 2009-11-14

**Authors:** Lukasz Porosa, Russell D. Viirre, Alan J. Lough

**Affiliations:** a Department of Chemistry and Biology, Ryerson University, Toronto, Ontario, Canada M5B 2K3; b Department of Chemistry, University of Toronto, 80 St George St, Toronto, Ontario, Canada M5B 2K3

## Abstract

In the title mol­ecule, C_27_H_26_Cl_2_N_2_O_2_, the chloro-substituted benzene rings make dihedral angles of 83.29 (9) and 80.81 (9)° with the benzene ring of the tetra­hydro­quinoline group. The dihedral angle formed by the two chloro-substituted benzene rings is 40.87 (12)°. The six-membered N-containing ring is in a half-chair conformation. In the crystal structure, inter­molecular N—H⋯O hydrogen bonds link mol­ecules into centrosymmetric dimers.

## Related literature

For the synthesis of the title compound, see: Porosa & Viirre (2009 ▶). For a related crystal structure, see: Wang *et al.* (2007 ▶)
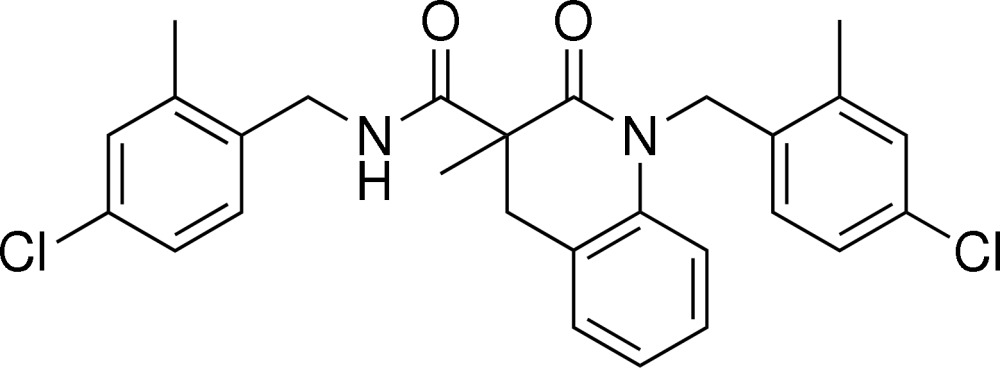


## Experimental

### 

#### Crystal data

C_27_H_26_Cl_2_N_2_O_2_
*M* *_r_* = 481.40
Triclinic, 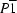

*a* = 10.1394 (6) Å
*b* = 10.7095 (6) Å
*c* = 12.2542 (4) Å
α = 82.084 (3)°
β = 71.403 (3)°
γ = 66.519 (2)°
*V* = 1156.66 (10) Å^3^
*Z* = 2
Mo *K*α radiation
μ = 0.31 mm^−1^
*T* = 150 K
0.20 × 0.12 × 0.10 mm

#### Data collection

Nonius KappaCCD diffractometer
Absorption correction: multi-scan from symmetry-related measurements (*SORTAV*; Blessing, 1995 ▶) *T*_min_ = 0.670, *T*_max_ = 0.974
10842 measured reflections
5170 independent reflections
2859 reflections with *I* > 2σ(*I*)
*R*_int_ = 0.067

#### Refinement

*R*[*F* ^2^ > 2σ(*F* ^2^)] = 0.064
*wR*(*F* ^2^) = 0.184
*S* = 1.02
5170 reflections
301 parameters
H-atom parameters constrained
Δρ_max_ = 0.42 e Å^−3^
Δρ_min_ = −0.30 e Å^−3^

### 

Data collection: *COLLECT* (Nonius, 2002 ▶); cell refinement: *DENZO-SMN* (Otwinowski & Minor, 1997 ▶); data reduction: *DENZO-SMN*; program(s) used to solve structure: *SIR92* (Altomare *et al.*, 1994 ▶); program(s) used to refine structure: *SHELXTL* (Sheldrick, 2008 ▶); molecular graphics: *PLATON* (Spek, 2009 ▶); software used to prepare material for publication: *SHELXTL*.

## Supplementary Material

Crystal structure: contains datablocks global, I. DOI: 10.1107/S1600536809046765/pv2230sup1.cif

Structure factors: contains datablocks I. DOI: 10.1107/S1600536809046765/pv2230Isup2.hkl

Additional supplementary materials:  crystallographic information; 3D view; checkCIF report

## Figures and Tables

**Table 1:** Hydrogen-bond geometry (Å, °)

*D*—H⋯*A*	*D*—H	H⋯*A*	*D*⋯*A*	*D*—H⋯*A*
N2—H1*N*⋯O1^i^	0.88	2.14	2.972 (3)	157

Symmetry code: (i) 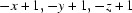.

**Fig. 1.**
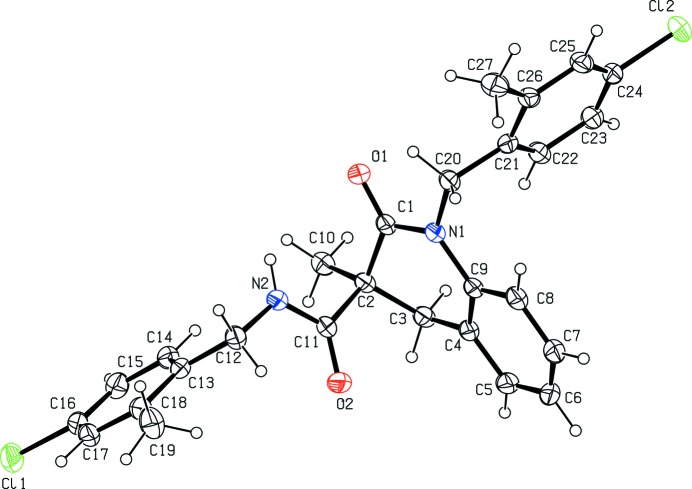
The molecular structure showing 30% probability displacement ellipsoids (arbitrary spheres for H atoms).

**Fig. 2.**
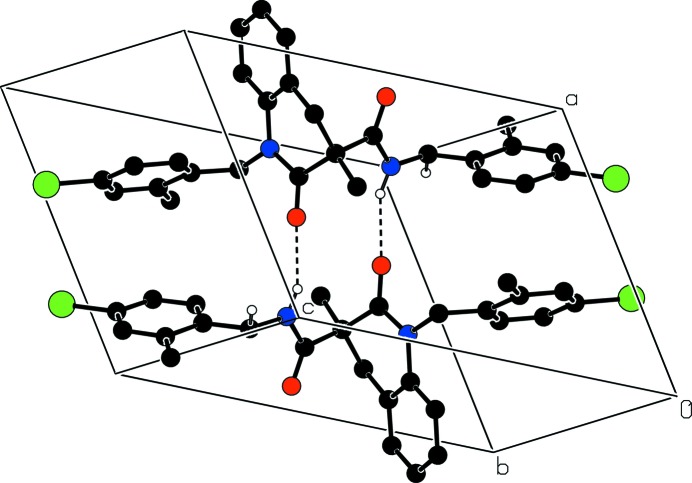
Part of the crystal structure showing hydrogen bonds as dashed lines.

**Fig. 3.**
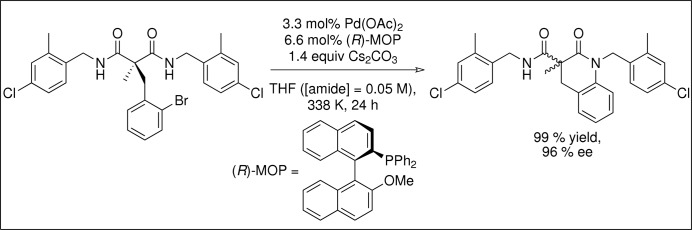
Reaction scheme.

## supplementary crystallographic information

### Comment

The title compound was prepared by an intramolecular Buchwald-Hartwig reaction of the corresponding malonamide under conditions we have previously described (Porosa & Viirre, 2009) (Fig. 3). The intention in this reaction was to preferentially arylate one of the two enantiotopic nitrogen atoms in the malonamide by exploiting the chiral influence of (*R*)-MOP ((*R*)-(+)-2-(diphenylphosphino)-2'-methoxy-1,1'-binaphthyl) as a catalyst component. Indeed, chiral HPLC analysis of the product indicated the highest enantioselectivity we have yet observed in this reaction, at 96% ee. It was hoped that the configuration of the major enantiomer could be determined from a crystal structure in order to correlate product and catalyst configuration and aid in the development of a mechanistic model for the reaction. The initially isolated product with 96% ee was a very viscous yellow oil. This was dissolved in diethyl ether and left to stand undisturbed at room temperature for several days. Upon evaporation of most of the solvent, a yellow oil was again obtained, but dispersed within it were small clear crystals. One of the single crystals was subjected to X-ray diffraction analysis and the crystal structure is reported herein.

The molecular structure of the title compound is shown in Fig. 1. To our surprise, it crystallized in a centrosymmetric space group. As there is no apparent mechanism by which the quaternary chiral center can epimerize, this demonstrates an impressive propensity for the racemate (essentially a 4% impurity in the initial product) to crystallize in preference to enantiopure material. In the title molecule, the C13—C18 and C21—C26 benzene rings form dihedral angles of 83.29 (9) and 80.81 (9)°, respectively with the C4—C9 benzene ring. The dihedral angle formed by the C13—C18 and C21—C26 benzene rings is 40.87 (12) °. The C1—C4/C9/N1 ring is in a half-chair conformation. In the crystal structure, intermolecular N—H···O hydrogen bonds link molecules into centrosymmetric dimers (Fig. 2).

Work is currently underway to crystallize enantiopure material.

### Experimental

The compound was prepared using the procedure previously described (Porosa & Viirre, 2009), using 2-(2-bromobenzyl)-*N*,*N*'-bis(4-chloro-2-methylbenzyl)-2-methylpropanediamide as a starting material. This material was recrystallized from diethylether to obtain small amounts of diffraction quality crystals of the title compound.

### Refinement

H atoms were placed in calculated positions with C—H distances in the range 0.95–0.99 Å; N—H = 0.88Å and included in the refinement in the riding-model approximation with *U*_iso_(H) = 1.2*U*_eq_(C,*N*) or *U*_iso_(H) = 1.5*U*_eq_(C) for methyl H atoms.

### Crystal data

**Table:**

C_27_H_26_Cl_2_N_2_O_2_	*Z* = 2
*M**_r_* = 481.40	*F*(000) = 504
Triclinic, *P*1	*D*_x_ = 1.382 Mg m^−^^3^
Hall symbol: -P 1	Mo *K*α radiation, λ = 0.71073 Å
*a* = 10.1394 (6) Å	Cell parameters from 10842 reflections
*b* = 10.7095 (6) Å	θ = 2.6–27.5°
*c* = 12.2542 (4) Å	µ = 0.31 mm^−^^1^
α = 82.084 (3)°	*T* = 150 K
β = 71.403 (3)°	Block, colourless
γ = 66.519 (2)°	0.20 × 0.12 × 0.10 mm
*V* = 1156.66 (10) Å^3^	

### Data collection

**Table:**

Nonius KappaCCD diffractometer	5170 independent reflections
Radiation source: fine-focus sealed tube	2859 reflections with *I* > 2σ(*I*)
graphite	*R*_int_ = 0.067
Detector resolution: 9 pixels mm^-1^	θ_max_ = 27.5°, θ_min_ = 2.6°
φ scans and ω scans with κ offsets	*h* = −12→13
Absorption correction: multi-scan from symmetry-related measurements (*SORTAV*; Blessing, 1995)	*k* = −13→13
*T*_min_ = 0.670, *T*_max_ = 0.974	*l* = −15→15
10842 measured reflections	

### Refinement

**Table:**

Refinement on *F*^2^	Primary atom site location: structure-invariant direct methods
Least-squares matrix: full	Secondary atom site location: difference Fourier map
*R*[*F*^2^ > 2σ(*F*^2^)] = 0.064	Hydrogen site location: inferred from neighbouring sites
*wR*(*F*^2^) = 0.184	H-atom parameters constrained
*S* = 1.02	*w* = 1/[σ^2^(*F*_o_^2^) + (0.0886*P*)^2^ + 0.0727*P*] where *P* = (*F*_o_^2^ + 2*F*_c_^2^)/3
5170 reflections	(Δ/σ)_max_ = 0.001
301 parameters	Δρ_max_ = 0.42 e Å^−^^3^
0 restraints	Δρ_min_ = −0.30 e Å^−^^3^

### Special details

**Table:**

Experimental. ^1^H NMR [400 MHz, CDCl3] δ_H_ 7.31–6.94 (m, 8H), 6.78 (dd, J = 2 Hz, J = 8 Hz, 1H), 6.63 (dd, J = 8 Hz, J = 14 Hz, 2H), 5.07–4.91 (m, 2H), 4.35 (d, J = 6 Hz, 2H), 3.52 (d, J = 16 Hz, 1H), 3.09 (d, J = 16 Hz, 1H), 2.36 (s, 3H), 2.15 (s, 3H), 1.57 (s, 3H). HRMS (EI-TOF) calculated for C_27_H_27_N_2_O_2_ (*M* + H)+ 481.1450; observed 481.1426. HPLC (Chiralcel OD—H column, eluting with 0.65 ml/min 10% i-PrOH:hexanes), tR minor = 20.8 min (peak area = 181909), tR major = 24.5 min (peak area = 9489431), enantiomer ratio = 98:2, 96% ee.
Geometry. All e.s.d.'s (except the e.s.d. in the dihedral angle between two l.s. planes) are estimated using the full covariance matrix. The cell e.s.d.'s are taken into account individually in the estimation of e.s.d.'s in distances, angles and torsion angles; correlations between e.s.d.'s in cell parameters are only used when they are defined by crystal symmetry. An approximate (isotropic) treatment of cell e.s.d.'s is used for estimating e.s.d.'s involving l.s. planes.
Refinement. Refinement of *F*^2^ against ALL reflections. The weighted *R*-factor *wR* and goodness of fit *S* are based on *F*^2^, conventional *R*-factors *R* are based on *F*, with *F* set to zero for negative *F*^2^. The threshold expression of *F*^2^ > σ(*F*^2^) is used only for calculating *R*-factors(gt) *etc*. and is not relevant to the choice of reflections for refinement. *R*-factors based on *F*^2^ are statistically about twice as large as those based on *F*, and *R*- factors based on ALL data will be even larger.

### Fractional atomic coordinates and isotropic or equivalent isotropic displacement parameters (Å^2^)

**Table:**

	*x*	*y*	*z*	*U*_iso_*/*U*_eq_	
Cl1	0.21891 (11)	0.99149 (9)	1.07942 (7)	0.0631 (3)	
Cl2	0.43748 (10)	0.28380 (10)	−0.14948 (6)	0.0707 (3)	
O1	0.4463 (2)	0.5129 (2)	0.39762 (16)	0.0514 (6)	
O2	0.0270 (2)	0.8179 (2)	0.61445 (16)	0.0507 (6)	
N1	0.2188 (3)	0.5470 (2)	0.37995 (18)	0.0405 (6)	
N2	0.2249 (3)	0.6438 (2)	0.65164 (18)	0.0424 (6)	
H1N	0.3172	0.5853	0.6235	0.051*	
C1	0.3161 (4)	0.5882 (3)	0.4071 (2)	0.0408 (7)	
C2	0.2518 (3)	0.7334 (3)	0.4536 (2)	0.0405 (7)	
C3	0.1526 (3)	0.8275 (3)	0.3812 (2)	0.0430 (7)	
H3A	0.1013	0.9195	0.4152	0.052*	
H3B	0.2167	0.8351	0.3025	0.052*	
C4	0.0378 (3)	0.7782 (3)	0.3742 (2)	0.0411 (7)	
C5	−0.1028 (4)	0.8655 (3)	0.3656 (2)	0.0497 (8)	
H5A	−0.1286	0.9611	0.3659	0.060*	
C6	−0.2068 (4)	0.8164 (4)	0.3567 (2)	0.0548 (9)	
H6A	−0.3029	0.8779	0.3516	0.066*	
C7	−0.1698 (4)	0.6789 (4)	0.3551 (2)	0.0474 (8)	
H7A	−0.2405	0.6453	0.3482	0.057*	
C8	−0.0307 (4)	0.5881 (3)	0.3635 (2)	0.0453 (8)	
H8A	−0.0058	0.4928	0.3619	0.054*	
C9	0.0724 (3)	0.6375 (3)	0.3742 (2)	0.0392 (7)	
C10	0.3780 (3)	0.7793 (3)	0.4494 (2)	0.0474 (8)	
H10A	0.3347	0.8697	0.4836	0.071*	
H10B	0.4370	0.7834	0.3692	0.071*	
H10C	0.4433	0.7143	0.4928	0.071*	
C11	0.1555 (3)	0.7353 (3)	0.5808 (2)	0.0411 (7)	
C12	0.1485 (4)	0.6406 (3)	0.7743 (2)	0.0430 (7)	
H12A	0.1870	0.5458	0.8029	0.052*	
H12B	0.0400	0.6670	0.7844	0.052*	
C13	0.1659 (3)	0.7323 (3)	0.8486 (2)	0.0391 (7)	
C14	0.2568 (3)	0.8058 (3)	0.8039 (2)	0.0436 (8)	
H14A	0.3098	0.7996	0.7242	0.052*	
C15	0.2724 (4)	0.8887 (3)	0.8728 (3)	0.0475 (8)	
H15A	0.3325	0.9413	0.8407	0.057*	
C16	0.1988 (4)	0.8925 (3)	0.9887 (3)	0.0448 (8)	
C17	0.1071 (4)	0.8214 (3)	1.0353 (2)	0.0443 (8)	
H17A	0.0565	0.8265	1.1155	0.053*	
C18	0.0882 (3)	0.7424 (3)	0.9662 (2)	0.0409 (7)	
C19	−0.0146 (4)	0.6665 (4)	1.0202 (3)	0.0611 (10)	
H19A	−0.0770	0.7029	1.0969	0.092*	
H19B	0.0457	0.5696	1.0270	0.092*	
H19C	−0.0793	0.6777	0.9719	0.092*	
C20	0.2704 (4)	0.4028 (3)	0.3528 (2)	0.0426 (7)	
H20A	0.1903	0.3687	0.3957	0.051*	
H20B	0.3596	0.3506	0.3801	0.051*	
C21	0.3107 (3)	0.3756 (3)	0.2262 (2)	0.0353 (7)	
C22	0.3173 (3)	0.4763 (3)	0.1428 (2)	0.0443 (8)	
H22A	0.2943	0.5657	0.1655	0.053*	
C23	0.3568 (3)	0.4491 (3)	0.0266 (2)	0.0454 (8)	
H23A	0.3613	0.5185	−0.0301	0.055*	
C24	0.3888 (3)	0.3208 (3)	−0.0038 (2)	0.0441 (8)	
C25	0.3860 (3)	0.2177 (3)	0.0767 (2)	0.0454 (8)	
H25A	0.4117	0.1284	0.0523	0.054*	
C26	0.3458 (3)	0.2435 (3)	0.1934 (2)	0.0387 (7)	
C27	0.3438 (4)	0.1298 (3)	0.2814 (3)	0.0522 (8)	
H27A	0.3800	0.0437	0.2417	0.078*	
H27B	0.2409	0.1496	0.3314	0.078*	
H27C	0.4092	0.1224	0.3280	0.078*	

### Atomic displacement parameters (Å^2^)

**Table:**

	*U*^11^	*U*^22^	*U*^33^	*U*^12^	*U*^13^	*U*^23^
Cl1	0.0762 (7)	0.0576 (6)	0.0658 (5)	−0.0194 (5)	−0.0375 (5)	−0.0109 (4)
Cl2	0.0653 (6)	0.0933 (7)	0.0363 (4)	−0.0069 (5)	−0.0133 (4)	−0.0228 (4)
O1	0.0427 (14)	0.0520 (14)	0.0461 (12)	0.0041 (11)	−0.0187 (10)	−0.0131 (10)
O2	0.0403 (13)	0.0555 (14)	0.0385 (11)	−0.0003 (11)	−0.0087 (9)	−0.0066 (9)
N1	0.0423 (15)	0.0379 (14)	0.0337 (12)	−0.0024 (12)	−0.0141 (11)	−0.0091 (10)
N2	0.0396 (14)	0.0446 (15)	0.0334 (12)	−0.0037 (12)	−0.0110 (10)	−0.0071 (10)
C1	0.045 (2)	0.0413 (18)	0.0271 (14)	−0.0046 (16)	−0.0121 (13)	−0.0058 (12)
C2	0.0418 (18)	0.0402 (17)	0.0308 (14)	−0.0047 (14)	−0.0098 (12)	−0.0083 (12)
C3	0.0456 (19)	0.0413 (18)	0.0332 (15)	−0.0070 (15)	−0.0096 (13)	−0.0066 (12)
C4	0.0429 (19)	0.0440 (19)	0.0269 (14)	−0.0046 (15)	−0.0116 (12)	−0.0037 (12)
C5	0.051 (2)	0.0443 (19)	0.0386 (16)	0.0018 (16)	−0.0163 (14)	−0.0055 (13)
C6	0.0394 (19)	0.074 (3)	0.0366 (16)	−0.0004 (18)	−0.0167 (14)	−0.0091 (15)
C7	0.044 (2)	0.061 (2)	0.0354 (15)	−0.0155 (18)	−0.0116 (13)	−0.0064 (14)
C8	0.050 (2)	0.050 (2)	0.0308 (14)	−0.0141 (17)	−0.0097 (13)	−0.0054 (12)
C9	0.0388 (18)	0.0443 (19)	0.0255 (13)	−0.0053 (15)	−0.0083 (12)	−0.0069 (12)
C10	0.0461 (19)	0.053 (2)	0.0396 (16)	−0.0152 (16)	−0.0089 (13)	−0.0086 (13)
C11	0.0432 (19)	0.0417 (18)	0.0369 (15)	−0.0083 (15)	−0.0149 (13)	−0.0115 (13)
C12	0.0493 (19)	0.0435 (18)	0.0362 (15)	−0.0140 (15)	−0.0160 (13)	−0.0026 (12)
C13	0.0329 (17)	0.0429 (17)	0.0357 (15)	−0.0049 (14)	−0.0137 (12)	−0.0021 (12)
C14	0.0375 (18)	0.053 (2)	0.0361 (15)	−0.0120 (16)	−0.0111 (13)	−0.0019 (13)
C15	0.0445 (19)	0.054 (2)	0.0483 (18)	−0.0209 (16)	−0.0182 (14)	0.0041 (14)
C16	0.0471 (19)	0.0397 (18)	0.0481 (17)	−0.0055 (16)	−0.0259 (15)	−0.0072 (13)
C17	0.051 (2)	0.0436 (18)	0.0330 (15)	−0.0102 (16)	−0.0142 (13)	−0.0029 (13)
C18	0.0430 (18)	0.0399 (18)	0.0361 (15)	−0.0122 (15)	−0.0124 (13)	0.0027 (12)
C19	0.074 (3)	0.075 (3)	0.0436 (18)	−0.039 (2)	−0.0172 (16)	0.0029 (16)
C20	0.0511 (19)	0.0363 (17)	0.0345 (14)	−0.0058 (15)	−0.0161 (13)	−0.0062 (12)
C21	0.0289 (16)	0.0395 (17)	0.0326 (14)	−0.0075 (13)	−0.0088 (11)	−0.0022 (12)
C22	0.0501 (19)	0.0404 (18)	0.0349 (15)	−0.0083 (15)	−0.0110 (13)	−0.0076 (13)
C23	0.0410 (18)	0.054 (2)	0.0331 (15)	−0.0115 (16)	−0.0091 (13)	0.0000 (13)
C24	0.0327 (17)	0.059 (2)	0.0344 (15)	−0.0081 (15)	−0.0097 (12)	−0.0116 (14)
C25	0.0397 (18)	0.0457 (19)	0.0499 (18)	−0.0116 (15)	−0.0105 (14)	−0.0184 (14)
C26	0.0295 (16)	0.0445 (19)	0.0399 (15)	−0.0111 (14)	−0.0069 (12)	−0.0098 (13)
C27	0.056 (2)	0.0424 (19)	0.0528 (19)	−0.0165 (17)	−0.0097 (15)	−0.0045 (14)

### Geometric parameters (Å, °)

**Table:**

Cl1—C16	1.744 (3)	C12—H12A	0.9900
Cl2—C24	1.750 (3)	C12—H12B	0.9900
O1—C1	1.219 (3)	C13—C14	1.381 (4)
O2—C11	1.220 (3)	C13—C18	1.400 (4)
N1—C1	1.369 (4)	C14—C15	1.389 (4)
N1—C9	1.432 (4)	C14—H14A	0.9500
N1—C20	1.468 (4)	C15—C16	1.375 (4)
N2—C11	1.347 (4)	C15—H15A	0.9500
N2—C12	1.458 (3)	C16—C17	1.370 (4)
N2—H1N	0.8800	C17—C18	1.379 (4)
C1—C2	1.539 (4)	C17—H17A	0.9500
C2—C3	1.526 (4)	C18—C19	1.507 (4)
C2—C10	1.528 (4)	C19—H19A	0.9800
C2—C11	1.551 (4)	C19—H19B	0.9800
C3—C4	1.486 (4)	C19—H19C	0.9800
C3—H3A	0.9900	C20—C21	1.511 (3)
C3—H3B	0.9900	C20—H20A	0.9900
C4—C5	1.387 (4)	C20—H20B	0.9900
C4—C9	1.404 (4)	C21—C22	1.387 (4)
C5—C6	1.390 (5)	C21—C26	1.398 (4)
C5—H5A	0.9500	C22—C23	1.389 (4)
C6—C7	1.369 (5)	C22—H22A	0.9500
C6—H6A	0.9500	C23—C24	1.357 (4)
C7—C8	1.385 (4)	C23—H23A	0.9500
C7—H7A	0.9500	C24—C25	1.379 (4)
C8—C9	1.392 (4)	C25—C26	1.390 (4)
C8—H8A	0.9500	C25—H25A	0.9500
C10—H10A	0.9800	C26—C27	1.513 (4)
C10—H10B	0.9800	C27—H27A	0.9800
C10—H10C	0.9800	C27—H27B	0.9800
C12—C13	1.519 (4)	C27—H27C	0.9800
			
C1—N1—C9	123.4 (2)	C14—C13—C18	118.7 (3)
C1—N1—C20	117.6 (2)	C14—C13—C12	122.1 (2)
C9—N1—C20	119.0 (2)	C18—C13—C12	119.2 (3)
C11—N2—C12	120.7 (2)	C13—C14—C15	121.5 (3)
C11—N2—H1N	119.7	C13—C14—H14A	119.2
C12—N2—H1N	119.7	C15—C14—H14A	119.2
O1—C1—N1	121.9 (3)	C16—C15—C14	118.3 (3)
O1—C1—C2	121.4 (3)	C16—C15—H15A	120.9
N1—C1—C2	116.6 (3)	C14—C15—H15A	120.9
C3—C2—C10	111.2 (2)	C17—C16—C15	121.5 (3)
C3—C2—C1	108.0 (2)	C17—C16—Cl1	118.5 (2)
C10—C2—C1	110.8 (2)	C15—C16—Cl1	120.0 (3)
C3—C2—C11	109.5 (2)	C16—C17—C18	120.1 (3)
C10—C2—C11	108.2 (2)	C16—C17—H17A	119.9
C1—C2—C11	109.1 (2)	C18—C17—H17A	119.9
C4—C3—C2	112.5 (2)	C17—C18—C13	119.8 (3)
C4—C3—H3A	109.1	C17—C18—C19	118.7 (3)
C2—C3—H3A	109.1	C13—C18—C19	121.5 (3)
C4—C3—H3B	109.1	C18—C19—H19A	109.5
C2—C3—H3B	109.1	C18—C19—H19B	109.5
H3A—C3—H3B	107.8	H19A—C19—H19B	109.5
C5—C4—C9	118.0 (3)	C18—C19—H19C	109.5
C5—C4—C3	122.7 (3)	H19A—C19—H19C	109.5
C9—C4—C3	119.3 (3)	H19B—C19—H19C	109.5
C4—C5—C6	121.5 (3)	N1—C20—C21	114.2 (2)
C4—C5—H5A	119.3	N1—C20—H20A	108.7
C6—C5—H5A	119.3	C21—C20—H20A	108.7
C7—C6—C5	119.5 (3)	N1—C20—H20B	108.7
C7—C6—H6A	120.2	C21—C20—H20B	108.7
C5—C6—H6A	120.2	H20A—C20—H20B	107.6
C6—C7—C8	120.9 (3)	C22—C21—C26	119.7 (2)
C6—C7—H7A	119.6	C22—C21—C20	122.0 (3)
C8—C7—H7A	119.6	C26—C21—C20	118.3 (2)
C7—C8—C9	119.4 (3)	C21—C22—C23	121.3 (3)
C7—C8—H8A	120.3	C21—C22—H22A	119.4
C9—C8—H8A	120.3	C23—C22—H22A	119.4
C8—C9—C4	120.7 (3)	C24—C23—C22	118.3 (3)
C8—C9—N1	121.1 (3)	C24—C23—H23A	120.8
C4—C9—N1	118.2 (3)	C22—C23—H23A	120.8
C2—C10—H10A	109.5	C23—C24—C25	121.9 (3)
C2—C10—H10B	109.5	C23—C24—Cl2	119.3 (2)
H10A—C10—H10B	109.5	C25—C24—Cl2	118.8 (2)
C2—C10—H10C	109.5	C24—C25—C26	120.5 (3)
H10A—C10—H10C	109.5	C24—C25—H25A	119.8
H10B—C10—H10C	109.5	C26—C25—H25A	119.8
O2—C11—N2	122.7 (3)	C25—C26—C21	118.3 (3)
O2—C11—C2	121.6 (3)	C25—C26—C27	120.3 (3)
N2—C11—C2	115.6 (2)	C21—C26—C27	121.4 (2)
N2—C12—C13	115.5 (2)	C26—C27—H27A	109.5
N2—C12—H12A	108.4	C26—C27—H27B	109.5
C13—C12—H12A	108.4	H27A—C27—H27B	109.5
N2—C12—H12B	108.4	C26—C27—H27C	109.5
C13—C12—H12B	108.4	H27A—C27—H27C	109.5
H12A—C12—H12B	107.5	H27B—C27—H27C	109.5

### Hydrogen-bond geometry (Å, °)

**Table:**

*D*—H···*A*	*D*—H	H···*A*	*D*···*A*	*D*—H···*A*
N2—H1N···O1^i^	0.88	2.14	2.972 (3)	157

Symmetry codes: (i) −*x*+1, −*y*+1, −*z*+1.
